# LiYbCl_4_(THF)_4_

**DOI:** 10.1107/S1600536811014395

**Published:** 2011-05-07

**Authors:** Lukas Richtera, Vojtech Jancik, Sona Hermanova, Karel Krpoun, Kimberly Thompson-Montero

**Affiliations:** a Institute of Materials Chemistry, Faculty of Chemistry, Brno University of Technology, Purkynova 118, 612 00 Brno, Czech Republic, and CEITEC BUT, Technicka 3058/10, 616 00, Czech Republic; b Centro Conjunto de Investigación en Química Sustentable UAEM-UNAM, Carr., Toluca-Atlacomulco Km 14.5, Toluca, Estado de México, 50200, México

## Abstract

The title compound, di-μ-chlorido-dichlorido-1κ^2^ *Cl*-tetra­kis­(tetra­hydro­furan)-1κ^2^ *O*,2κ^2^ *O*-lithiumytterbium(III), [LiYbCl_4_(C_4_H_8_O)_4_], was prepared by the reaction of YbCl_3_(THF)_3_ with LiCl in THF (THF is tetra­hydro­furan). The central motif of the structure is a Yb(μ-Cl)_2_Li ring. The Yb atom is hexa­coordinated to four Cl atoms and two THF mol­ecules oriented in a *trans* fashion. The Li atom has a tetra­hedral environment and is coordinated to two Cl atoms and two THF mol­ecules. No inter­molecular inter­actions other than van der Waals forces were observed. Two of the THF mol­ecules are disordered over two positions.

## Related literature

For the isotypic yttrium compound, see Mingqing *et al.* (1986 ▶). For similar lithium compounds with other trivalent cations, see: Chitsaz *et al.* (2001 ▶) for V^III^; Neumüller *et al.* (1996 ▶) for Ti^III^; McGuinness *et al.* (2006 ▶) for Cr^III^.
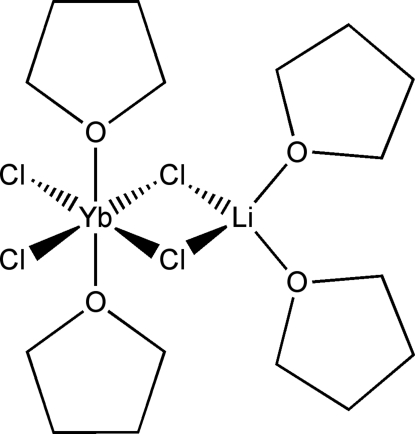


## Experimental

### 

#### Crystal data

[LiYbCl_4_(C_4_H_8_O)_4_]
*M* *_r_* = 610.20
Monoclinic, 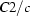

*a* = 20.9150 (14) Å
*b* = 10.1565 (7) Å
*c* = 21.8810 (14) Å
β = 91.376 (1)°
*V* = 4646.7 (5) Å^3^
*Z* = 8
Mo *K*α radiation
μ = 4.50 mm^−1^
*T* = 100 K
0.37 × 0.16 × 0.14 mm

#### Data collection

Bruker APEXII CCD diffractometer
Absorption correction: multi-scan (*SADABS*; Sheldrick, 1996 ▶) *T*_min_ = 0.430, *T*_max_ = 0.543
17457 measured reflections
4040 independent reflections
3804 reflections with *I* > 2σ(*I*)
*R*_int_ = 0.020

#### Refinement

*R*[*F* ^2^ > 2σ(*F* ^2^)] = 0.023
*wR*(*F* ^2^) = 0.050
*S* = 1.22
4040 reflections
302 parameters
388 restraints
H-atom parameters constrained
Δρ_max_ = 1.06 e Å^−3^
Δρ_min_ = −1.81 e Å^−3^

### 

Data collection: *APEX2* (Bruker, 2004 ▶); cell refinement: *SAINT* (Bruker, 2004 ▶); data reduction: *SAINT*; program(s) used to solve structure: *SHELXS97* (Sheldrick, 2008 ▶); program(s) used to refine structure: *SHELXL97* (Sheldrick, 2008 ▶); molecular graphics: *GRETEP* (Laugier & Bochu, 2003 ▶); software used to prepare material for publication: *SHELXTL* (Sheldrick, 2008 ▶).

## Supplementary Material

Crystal structure: contains datablocks I, global. DOI: 10.1107/S1600536811014395/fi2106sup1.cif

Structure factors: contains datablocks I. DOI: 10.1107/S1600536811014395/fi2106Isup2.hkl

Additional supplementary materials:  crystallographic information; 3D view; checkCIF report

## Figures and Tables

**Fig. 1.**
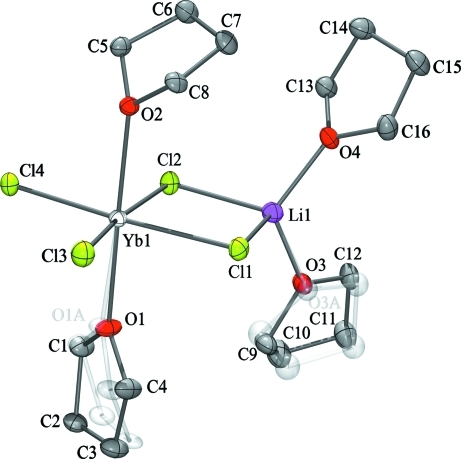
Crystal structure of LiYbCl4(THF)4 with thermal ellipsoids at 50% probability. All hydrogen atoms have been omitted for clarity. Transparent parts belong to the minor orientation of the disordered THF molecules.

**Fig. 2.**
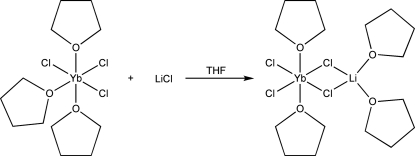
, Preparation of LiYbCl4(THF)4.

## supplementary crystallographic information

### Comment

Lanthanide compounds containing small ligands can easily form adducts or complex structures with electron donating ligands such as oxygen, halogenides *etc*. It is well known, that in presence of a Lewis acid such as lithium, mixed metal complexes can be formed. Thus, the reaction of YbCl_3_(THF)_3_ with LiCl in THF resulted in the formation of LiYbCl_4_(THF)_4_ as the only product (Scheme 1). Two types of chlorine atoms can be observed in the molecule: two terminal ones, which are coordinated to the ytterbium atom, whereas the other two are bridging the ytterbium and lithium atoms. The coordination spheres of the metals are completed by four THF molecules (two per metal) resulting in a hexacoordinated octahedral environment on the ytterbium atom and tetrahedral coordination on the lithium atom. The THF molecules on the ytterbium center are oriented *trans* to each other with an angle of 179.0 (5)° (for the major position of O1). The values for the *cis* Cl—Yb—Cl angles show high variation (82.95 (3) – 99.06 (3)°) due to the coordination of Cl1 and Cl2 to the lithium center, which results also in 172.39 (3)° and 171.11 (3)° *trans* Cl—Yb—Cl angles. The tetrahedron arround the lithium atom is also disordered with the angles ranging from 94.9 (2) to 114.2 (3)°. The molecular structure of LiYbCl_4_(THF)_4_ is depicted in Figure 1.

### Experimental

YbCl_3_(THF)_3_ (200 mg, 0.4 mmol) and LiCl (25.7 mg, 0.6 mmol) were mixed as solids in a schlenk flask and THF (10 ml) was added at ambient temperature. The mixture was stirred for 4 h. During this time, both components dissolved completely. The resulting solution was filtered and the solvent evaporated under vacuum to dryness. The product was obtained in a form of transparent colourless crystals in 86% yield. Single crystals were obtained from a saturated THF solution at -30 °C.

### Refinement

The hydrogen atoms were placed at calculated positions (*H*—CMethylene = 0.99 Å) and were refined with *U_ij_* set to 1.2 of the parent carbon atom. The SIMU, DELU and SAME restraints in *SHELXL97* were used in the refinement of the two disordered THF molecules (O1—C4 and O1a—C4a: occupancies 63 (2):37 (2), O3—C12 and O3a—C12*a*: occupancies 65 (2):35). The maximum/minimum of the difference electron density is found 1.10 and 1.31Å, respectively, from Li1.

### Crystal data

**Table:**

[LiYbCl_4_(C_4_H_8_O)_4_]	*F*(000) = 2408
*M**_r_* = 610.20	*D*_x_ = 1.744 Mg m^−^^3^
Monoclinic, *C*2/*c*	Mo *K*α radiation, λ = 0.71073 Å
Hall symbol: -C2yc	Cell parameters from 9956 reflections
*a* = 20.9150 (14) Å	θ = 2.2–25.0°
*b* = 10.1565 (7) Å	µ = 4.50 mm^−^^1^
*c* = 21.8810 (14) Å	*T* = 100 K
β = 91.376 (1)°	Prism, colourless
*V* = 4646.7 (5) Å^3^	0.37 × 0.16 × 0.14 mm
*Z* = 8	

### Data collection

**Table:**

Bruker APEXII CCD diffractometer	4040 independent reflections
Radiation source: fine-focus sealed tube	3804 reflections with *I* > 2σ(*I*)
graphite	*R*_int_ = 0.020
Detector resolution: 8.333 pixels mm^-1^	θ_max_ = 25.0°, θ_min_ = 2.2°
ω scans	*h* = −24→24
Absorption correction: multi-scan (*SADABS*; Sheldrick, 1996)	*k* = −12→12
*T*_min_ = 0.430, *T*_max_ = 0.543	*l* = −25→26
17457 measured reflections	

### Refinement

**Table:**

Refinement on *F*^2^	Primary atom site location: structure-invariant direct methods
Least-squares matrix: full	Secondary atom site location: difference Fourier map
*R*[*F*^2^ > 2σ(*F*^2^)] = 0.023	Hydrogen site location: inferred from neighbouring sites
*wR*(*F*^2^) = 0.050	H-atom parameters constrained
*S* = 1.22	*w* = 1/[σ^2^(*F*_o_^2^) + (0.0069*P*)^2^ + 33.1382*P*] where *P* = (*F*_o_^2^ + 2*F*_c_^2^)/3
4040 reflections	(Δ/σ)_max_ = 0.001
302 parameters	Δρ_max_ = 1.06 e Å^−^^3^
388 restraints	Δρ_min_ = −1.81 e Å^−^^3^

### Special details

**Table:**

Geometry. All e.s.d.'s (except the e.s.d. in the dihedral angle between two l.s. planes) are estimated using the full covariance matrix. The cell e.s.d.'s are taken into account individually in the estimation of e.s.d.'s in distances, angles and torsion angles; correlations between e.s.d.'s in cell parameters are only used when they are defined by crystal symmetry. An approximate (isotropic) treatment of cell e.s.d.'s is used for estimating e.s.d.'s involving l.s. planes.
Refinement. Refinement of *F*^2^ against ALL reflections. The weighted *R*-factor *wR* and goodness of fit *S* are based on *F*^2^, conventional *R*-factors *R* are based on *F*, with *F* set to zero for negative *F*^2^. The threshold expression of *F*^2^ > σ(*F*^2^) is used only for calculating *R*-factors(gt) *etc*. and is not relevant to the choice of reflections for refinement. *R*-factors based on *F*^2^ are statistically about twice as large as those based on *F*, and *R*- factors based on ALL data will be even larger.

### Fractional atomic coordinates and isotropic or equivalent isotropic displacement parameters (Å^2^)

**Table:**

	*x*	*y*	*z*	*U*_iso_*/*U*_eq_	Occ. (<1)
Yb1	0.133434 (8)	0.593279 (16)	0.364780 (7)	0.01390 (6)	
Li1	0.1330 (3)	0.9443 (7)	0.3598 (3)	0.0212 (14)	
Cl1	0.21554 (4)	0.78897 (10)	0.36461 (4)	0.0200 (2)	
Cl2	0.04911 (4)	0.78493 (9)	0.35817 (4)	0.0197 (2)	
Cl3	0.22670 (5)	0.43634 (10)	0.37626 (5)	0.0259 (2)	
Cl4	0.04294 (5)	0.42804 (10)	0.36458 (4)	0.0205 (2)	
O1	0.1404 (6)	0.591 (2)	0.2613 (3)	0.0222 (17)	0.633 (19)
C1	0.0864 (5)	0.586 (3)	0.2182 (5)	0.0238 (14)	0.633 (19)
H1A	0.0626	0.6701	0.2184	0.029*	0.633 (19)
H1B	0.0569	0.5138	0.2289	0.029*	0.633 (19)
C2	0.1150 (4)	0.5616 (12)	0.1559 (3)	0.0250 (16)	0.633 (19)
H2A	0.1085	0.4689	0.1433	0.030*	0.633 (19)
H2B	0.0945	0.6195	0.1247	0.030*	0.633 (19)
C3	0.1828 (4)	0.5909 (13)	0.1622 (4)	0.0313 (19)	0.633 (19)
H3A	0.1920	0.6782	0.1442	0.038*	0.633 (19)
H3B	0.2080	0.5238	0.1406	0.038*	0.633 (19)
C4	0.2004 (5)	0.5907 (13)	0.2291 (4)	0.0283 (19)	0.633 (19)
H4A	0.2258	0.5114	0.2398	0.034*	0.633 (19)
H4B	0.2259	0.6698	0.2399	0.034*	0.633 (19)
O1A	0.1374 (11)	0.578 (4)	0.2622 (5)	0.023 (2)	0.367 (19)
C1A	0.0833 (8)	0.585 (4)	0.2190 (8)	0.024 (2)	0.367 (19)
H1A1	0.0518	0.6508	0.2325	0.028*	0.367 (19)
H1A2	0.0618	0.4982	0.2155	0.028*	0.367 (19)
C2A	0.1111 (6)	0.625 (2)	0.1583 (6)	0.027 (2)	0.367 (19)
H2A1	0.0983	0.5610	0.1260	0.033*	0.367 (19)
H2A2	0.0955	0.7131	0.1461	0.033*	0.367 (19)
C3A	0.1810 (6)	0.6256 (19)	0.1670 (7)	0.027 (2)	0.367 (19)
H3A1	0.1972	0.7172	0.1683	0.032*	0.367 (19)
H3A2	0.2016	0.5786	0.1331	0.032*	0.367 (19)
C4A	0.1956 (9)	0.557 (2)	0.2273 (8)	0.026 (2)	0.367 (19)
H4A1	0.2036	0.4617	0.2210	0.031*	0.367 (19)
H4A2	0.2333	0.5963	0.2484	0.031*	0.367 (19)
O2	0.12665 (11)	0.5996 (3)	0.46880 (11)	0.0162 (5)	
O4	0.13057 (13)	1.0584 (3)	0.42837 (12)	0.0201 (6)	
C5	0.06770 (16)	0.6233 (4)	0.50160 (17)	0.0188 (8)	
H5A	0.0590	0.5499	0.5300	0.023*	
H5B	0.0309	0.6329	0.4727	0.023*	
C6	0.07979 (17)	0.7510 (4)	0.53669 (18)	0.0235 (9)	
H6A	0.0557	0.8246	0.5175	0.028*	
H6B	0.0665	0.7420	0.5796	0.028*	
C7	0.15103 (17)	0.7754 (4)	0.53396 (18)	0.0222 (9)	
H7A	0.1693	0.7958	0.5750	0.027*	
H7B	0.1603	0.8493	0.5060	0.027*	
C8	0.17804 (16)	0.6469 (4)	0.50985 (16)	0.0185 (8)	
H8A	0.2179	0.6621	0.4874	0.022*	
H8B	0.1867	0.5836	0.5435	0.022*	
O3	0.1302 (6)	1.0384 (7)	0.2846 (2)	0.0238 (14)	0.65 (3)
C9	0.1282 (7)	0.9709 (9)	0.2256 (4)	0.0283 (17)	0.65 (3)
H9A	0.1126	0.8794	0.2295	0.034*	0.65 (3)
H9B	0.1706	0.9704	0.2066	0.034*	0.65 (3)
C10	0.0797 (7)	1.0583 (9)	0.1892 (4)	0.0330 (17)	0.65 (3)
H10A	0.0854	1.0502	0.1446	0.040*	0.65 (3)
H10B	0.0350	1.0362	0.1990	0.040*	0.65 (3)
C11	0.0979 (8)	1.1940 (8)	0.2121 (4)	0.0320 (17)	0.65 (3)
H11A	0.1369	1.2265	0.1922	0.038*	0.65 (3)
H11B	0.0627	1.2577	0.2048	0.038*	0.65 (3)
C12	0.1097 (6)	1.1718 (8)	0.2783 (4)	0.0249 (16)	0.65 (3)
H12A	0.1431	1.2325	0.2943	0.030*	0.65 (3)
H12B	0.0700	1.1866	0.3011	0.030*	0.65 (3)
O3A	0.1437 (8)	1.0361 (14)	0.2847 (4)	0.021 (2)*	0.35 (3)
C9A	0.1190 (13)	0.9661 (13)	0.2316 (9)	0.031 (3)*	0.35 (3)
H9C	0.0821	0.9110	0.2430	0.038*	0.35 (3)
H9D	0.1525	0.9078	0.2154	0.038*	0.35 (3)
C10A	0.0989 (12)	1.0617 (16)	0.1847 (6)	0.032 (3)*	0.35 (3)
H10C	0.0521	1.0568	0.1771	0.038*	0.35 (3)
H10D	0.1206	1.0437	0.1458	0.038*	0.35 (3)
C11A	0.1174 (12)	1.1948 (13)	0.2088 (6)	0.027 (3)*	0.35 (3)
H11C	0.0833	1.2602	0.1998	0.033*	0.35 (3)
H11D	0.1577	1.2254	0.1907	0.033*	0.35 (3)
C12A	0.1259 (12)	1.1742 (15)	0.2772 (6)	0.026 (3)*	0.35 (3)
H12C	0.1599	1.2323	0.2941	0.031*	0.35 (3)
H12D	0.0856	1.1932	0.2984	0.031*	0.35 (3)
C13	0.07086 (19)	1.1028 (4)	0.45334 (18)	0.0230 (9)	
H13A	0.0397	1.0297	0.4551	0.028*	
H13B	0.0521	1.1749	0.4283	0.028*	
C14	0.0885 (2)	1.1506 (5)	0.51643 (19)	0.0295 (10)	
H14A	0.0932	1.0765	0.5456	0.035*	
H14B	0.0564	1.2134	0.5317	0.035*	
C15	0.1523 (2)	1.2180 (5)	0.5061 (2)	0.0284 (10)	
H15A	0.1789	1.2198	0.5441	0.034*	
H15B	0.1461	1.3093	0.4912	0.034*	
C16	0.18227 (19)	1.1318 (4)	0.45774 (19)	0.0241 (9)	
H16A	0.2043	1.1868	0.4274	0.029*	
H16B	0.2138	1.0709	0.4768	0.029*	

### Atomic displacement parameters (Å^2^)

**Table:**

	*U*^11^	*U*^22^	*U*^33^	*U*^12^	*U*^13^	*U*^23^
Yb1	0.01507 (9)	0.01596 (9)	0.01064 (8)	−0.00031 (7)	−0.00027 (6)	0.00006 (7)
Li1	0.025 (3)	0.023 (3)	0.016 (3)	0.001 (3)	−0.001 (3)	0.004 (3)
Cl1	0.0172 (5)	0.0218 (5)	0.0209 (5)	−0.0027 (4)	−0.0016 (4)	0.0032 (4)
Cl2	0.0172 (5)	0.0192 (5)	0.0226 (5)	0.0024 (4)	−0.0014 (4)	0.0012 (4)
Cl3	0.0242 (5)	0.0266 (6)	0.0268 (5)	0.0070 (4)	0.0018 (4)	0.0045 (4)
Cl4	0.0217 (5)	0.0181 (5)	0.0218 (5)	−0.0037 (4)	0.0001 (4)	−0.0014 (4)
O1	0.016 (2)	0.039 (4)	0.012 (2)	−0.004 (3)	0.0000 (18)	0.000 (2)
C1	0.021 (3)	0.037 (3)	0.014 (3)	−0.006 (3)	−0.004 (2)	−0.001 (3)
C2	0.032 (3)	0.031 (4)	0.012 (2)	−0.004 (3)	−0.001 (2)	−0.003 (3)
C3	0.032 (3)	0.045 (5)	0.017 (3)	0.012 (3)	0.007 (2)	−0.003 (3)
C4	0.019 (3)	0.047 (5)	0.019 (3)	−0.010 (3)	0.006 (2)	−0.008 (3)
O1A	0.018 (4)	0.039 (5)	0.012 (3)	−0.004 (4)	0.002 (3)	−0.004 (3)
C1A	0.021 (3)	0.037 (4)	0.013 (4)	−0.005 (4)	−0.003 (3)	−0.001 (4)
C2A	0.030 (3)	0.039 (5)	0.013 (3)	−0.005 (4)	−0.001 (3)	−0.004 (4)
C3A	0.028 (3)	0.038 (5)	0.014 (3)	0.001 (4)	0.010 (3)	−0.008 (4)
C4A	0.021 (3)	0.040 (5)	0.017 (4)	−0.003 (4)	0.003 (3)	−0.008 (4)
O2	0.0151 (13)	0.0200 (14)	0.0135 (13)	−0.0018 (11)	−0.0001 (10)	−0.0004 (11)
O4	0.0185 (14)	0.0201 (15)	0.0217 (14)	0.0017 (11)	0.0018 (11)	−0.0065 (12)
C5	0.0158 (19)	0.024 (2)	0.0162 (19)	−0.0029 (16)	0.0027 (15)	−0.0007 (16)
C6	0.019 (2)	0.028 (2)	0.023 (2)	−0.0008 (18)	0.0028 (17)	−0.0055 (18)
C7	0.021 (2)	0.025 (2)	0.020 (2)	−0.0004 (18)	−0.0045 (16)	−0.0040 (17)
C8	0.016 (2)	0.026 (2)	0.0131 (18)	−0.0010 (16)	−0.0050 (15)	−0.0023 (16)
O3	0.033 (3)	0.019 (2)	0.0188 (19)	0.007 (2)	0.0012 (18)	−0.0023 (15)
C9	0.045 (4)	0.023 (3)	0.018 (3)	−0.001 (3)	0.009 (3)	−0.003 (2)
C10	0.036 (4)	0.035 (3)	0.028 (3)	−0.004 (3)	−0.008 (3)	−0.002 (2)
C11	0.037 (4)	0.027 (3)	0.032 (3)	0.000 (3)	−0.007 (3)	0.008 (2)
C12	0.028 (4)	0.018 (2)	0.029 (3)	0.003 (3)	−0.003 (3)	−0.002 (2)
C13	0.021 (2)	0.026 (2)	0.022 (2)	0.0020 (18)	0.0034 (16)	−0.0034 (18)
C14	0.029 (2)	0.038 (3)	0.021 (2)	0.005 (2)	0.0057 (18)	−0.004 (2)
C15	0.028 (2)	0.031 (2)	0.027 (2)	0.001 (2)	−0.0027 (18)	−0.0097 (19)
C16	0.020 (2)	0.023 (2)	0.030 (2)	−0.0005 (17)	0.0008 (17)	−0.0033 (18)

### Geometric parameters (Å, °)

**Table:**

Yb1—O1A	2.254 (12)	C6—C7	1.513 (5)
Yb1—O1	2.273 (7)	C6—H6A	0.9900
Yb1—O2	2.285 (2)	C6—H6B	0.9900
Yb1—Cl3	2.5270 (10)	C7—C8	1.522 (5)
Yb1—Cl4	2.5294 (10)	C7—H7A	0.9900
Yb1—Cl1	2.6266 (10)	C7—H7B	0.9900
Yb1—Cl2	2.6283 (9)	C8—H8A	0.9900
Li1—O4	1.898 (7)	C8—H8B	0.9900
Li1—O3	1.904 (7)	O3—C12	1.426 (9)
Li1—O3A	1.907 (8)	O3—C9	1.460 (9)
Li1—Cl1	2.338 (7)	C9—C10	1.553 (14)
Li1—Cl2	2.387 (7)	C9—H9A	0.9900
O1—C4	1.453 (5)	C9—H9B	0.9900
O1—C1	1.455 (5)	C10—C11	1.512 (11)
C1—C2	1.522 (7)	C10—H10A	0.9900
C1—H1A	0.9900	C10—H10B	0.9900
C1—H1B	0.9900	C11—C12	1.482 (10)
C2—C3	1.453 (7)	C11—H11A	0.9900
C2—H2A	0.9900	C11—H11B	0.9900
C2—H2B	0.9900	C12—H12A	0.9900
C3—C4	1.501 (6)	C12—H12B	0.9900
C3—H3A	0.9900	O3A—C9A	1.448 (10)
C3—H3B	0.9900	O3A—C12A	1.459 (10)
C4—H4A	0.9900	C9A—C10A	1.466 (10)
C4—H4B	0.9900	C9A—H9C	0.9900
O1A—C1A	1.459 (7)	C9A—H9D	0.9900
O1A—C4A	1.468 (7)	C10A—C11A	1.499 (10)
C1A—C2A	1.517 (8)	C10A—H10C	0.9900
C1A—H1A1	0.9900	C10A—H10D	0.9900
C1A—H1A2	0.9900	C11A—C12A	1.517 (10)
C2A—C3A	1.472 (8)	C11A—H11C	0.9900
C2A—H2A1	0.9900	C11A—H11D	0.9900
C2A—H2A2	0.9900	C12A—H12C	0.9900
C3A—C4A	1.517 (8)	C12A—H12D	0.9900
C3A—H3A1	0.9900	C13—C14	1.501 (6)
C3A—H3A2	0.9900	C13—H13A	0.9900
C4A—H4A1	0.9900	C13—H13B	0.9900
C4A—H4A2	0.9900	C14—C15	1.521 (6)
O2—C5	1.461 (4)	C14—H14A	0.9900
O2—C8	1.465 (4)	C14—H14B	0.9900
O4—C13	1.447 (5)	C15—C16	1.521 (6)
O4—C16	1.451 (5)	C15—H15A	0.9900
C5—C6	1.526 (5)	C15—H15B	0.9900
C5—H5A	0.9900	C16—H16A	0.9900
C5—H5B	0.9900	C16—H16B	0.9900
			
O1A—Yb1—O1	3.7 (13)	C16—O4—Li1	129.1 (3)
O1A—Yb1—O2	177.2 (9)	O2—C5—C6	104.8 (3)
O1—Yb1—O2	179.0 (5)	O2—C5—H5A	110.8
O1A—Yb1—Cl3	90.5 (8)	C6—C5—H5A	110.8
O1—Yb1—Cl3	91.4 (4)	O2—C5—H5B	110.8
O2—Yb1—Cl3	89.14 (7)	C6—C5—H5B	110.8
O1A—Yb1—Cl4	89.8 (8)	H5A—C5—H5B	108.9
O1—Yb1—Cl4	93.3 (4)	C7—C6—C5	105.7 (3)
O2—Yb1—Cl4	87.48 (6)	C7—C6—H6A	110.6
Cl3—Yb1—Cl4	99.06 (3)	C5—C6—H6A	110.6
O1A—Yb1—Cl1	90.7 (8)	C7—C6—H6B	110.6
O1—Yb1—Cl1	87.1 (4)	C5—C6—H6B	110.6
O2—Yb1—Cl1	92.09 (7)	H6A—C6—H6B	108.7
Cl3—Yb1—Cl1	88.53 (3)	C6—C7—C8	104.3 (3)
Cl4—Yb1—Cl1	172.39 (3)	C6—C7—H7A	110.9
O1A—Yb1—Cl2	92.2 (8)	C8—C7—H7A	110.9
O1—Yb1—Cl2	90.7 (5)	C6—C7—H7B	110.9
O2—Yb1—Cl2	88.64 (7)	C8—C7—H7B	110.9
Cl3—Yb1—Cl2	171.11 (3)	H7A—C7—H7B	108.9
Cl4—Yb1—Cl2	89.44 (3)	O2—C8—C7	102.7 (3)
Cl1—Yb1—Cl2	82.95 (3)	O2—C8—H8A	111.2
O1A—Yb1—Li1	92.3 (9)	C7—C8—H8A	111.2
O1—Yb1—Li1	88.9 (5)	O2—C8—H8B	111.2
O2—Yb1—Li1	90.12 (12)	C7—C8—H8B	111.2
Cl3—Yb1—Li1	129.40 (11)	H8A—C8—H8B	109.1
Cl4—Yb1—Li1	131.45 (11)	C12—O3—C9	111.0 (5)
Cl1—Yb1—Li1	40.95 (11)	C12—O3—Li1	124.3 (6)
Cl2—Yb1—Li1	42.01 (11)	C9—O3—Li1	121.8 (6)
O4—Li1—O3	112.1 (4)	O3—C9—C10	100.9 (9)
O4—Li1—O3A	112.8 (6)	O3—C9—H9A	111.6
O3—Li1—O3A	8.5 (6)	C10—C9—H9A	111.6
O4—Li1—Cl1	114.2 (3)	O3—C9—H9B	111.6
O3—Li1—Cl1	112.7 (4)	C10—C9—H9B	111.6
O3A—Li1—Cl1	105.5 (6)	H9A—C9—H9B	109.4
O4—Li1—Cl2	113.1 (3)	C11—C10—C9	101.2 (6)
O3—Li1—Cl2	108.6 (4)	C11—C10—H10A	111.5
O3A—Li1—Cl2	114.8 (6)	C9—C10—H10A	111.5
Cl1—Li1—Cl2	94.9 (2)	C11—C10—H10B	111.5
O4—Li1—Yb1	125.9 (3)	C9—C10—H10B	111.5
O3—Li1—Yb1	121.9 (4)	H10A—C10—H10B	109.3
O3A—Li1—Yb1	121.0 (6)	C12—C11—C10	102.7 (6)
Cl1—Li1—Yb1	47.41 (12)	C12—C11—H11A	111.2
Cl2—Li1—Yb1	47.47 (12)	C10—C11—H11A	111.2
Li1—Cl1—Yb1	91.65 (17)	C12—C11—H11B	111.2
Li1—Cl2—Yb1	90.52 (17)	C10—C11—H11B	111.2
C4—O1—C1	110.6 (4)	H11A—C11—H11B	109.1
C4—O1—Yb1	124.0 (7)	O3—C12—C11	106.3 (6)
C1—O1—Yb1	125.3 (7)	O3—C12—H12A	110.5
O1—C1—C2	105.7 (4)	C11—C12—H12A	110.5
O1—C1—H1A	110.6	O3—C12—H12B	110.5
C2—C1—H1A	110.6	C11—C12—H12B	110.5
O1—C1—H1B	110.6	H12A—C12—H12B	108.7
C2—C1—H1B	110.6	C9A—O3A—C12A	107.2 (7)
H1A—C1—H1B	108.7	C9A—O3A—Li1	113.9 (11)
C3—C2—C1	106.5 (4)	C12A—O3A—Li1	122.2 (11)
C3—C2—H2A	110.4	O3A—C9A—C10A	109.1 (6)
C1—C2—H2A	110.4	O3A—C9A—H9C	109.9
C3—C2—H2B	110.4	C10A—C9A—H9C	109.9
C1—C2—H2B	110.4	O3A—C9A—H9D	109.9
H2A—C2—H2B	108.6	C10A—C9A—H9D	109.9
C2—C3—C4	108.0 (4)	H9C—C9A—H9D	108.3
C2—C3—H3A	110.1	C9A—C10A—C11A	106.4 (6)
C4—C3—H3A	110.1	C9A—C10A—H10C	110.4
C2—C3—H3B	110.1	C11A—C10A—H10C	110.4
C4—C3—H3B	110.1	C9A—C10A—H10D	110.4
H3A—C3—H3B	108.4	C11A—C10A—H10D	110.4
O1—C4—C3	106.1 (4)	H10C—C10A—H10D	108.6
O1—C4—H4A	110.5	C10A—C11A—C12A	104.3 (7)
C3—C4—H4A	110.5	C10A—C11A—H11C	110.9
O1—C4—H4B	110.5	C12A—C11A—H11C	110.9
C3—C4—H4B	110.5	C10A—C11A—H11D	110.9
H4A—C4—H4B	108.7	C12A—C11A—H11D	110.9
C1A—O1A—C4A	108.1 (6)	H11C—C11A—H11D	108.9
C1A—O1A—Yb1	126.5 (13)	O3A—C12A—C11A	105.5 (6)
C4A—O1A—Yb1	125.4 (12)	O3A—C12A—H12C	110.6
O1A—C1A—C2A	105.8 (5)	C11A—C12A—H12C	110.6
O1A—C1A—H1A1	110.6	O3A—C12A—H12D	110.6
C2A—C1A—H1A1	110.6	C11A—C12A—H12D	110.6
O1A—C1A—H1A2	110.6	H12C—C12A—H12D	108.8
C2A—C1A—H1A2	110.6	O4—C13—C14	104.6 (3)
H1A1—C1A—H1A2	108.7	O4—C13—H13A	110.8
C3A—C2A—C1A	106.8 (4)	C14—C13—H13A	110.8
C3A—C2A—H2A1	110.4	O4—C13—H13B	110.8
C1A—C2A—H2A1	110.4	C14—C13—H13B	110.8
C3A—C2A—H2A2	110.4	H13A—C13—H13B	108.9
C1A—C2A—H2A2	110.4	C13—C14—C15	101.9 (3)
H2A1—C2A—H2A2	108.6	C13—C14—H14A	111.4
C2A—C3A—C4A	106.7 (5)	C15—C14—H14A	111.4
C2A—C3A—H3A1	110.4	C13—C14—H14B	111.4
C4A—C3A—H3A1	110.4	C15—C14—H14B	111.4
C2A—C3A—H3A2	110.4	H14A—C14—H14B	109.2
C4A—C3A—H3A2	110.4	C14—C15—C16	102.8 (3)
H3A1—C3A—H3A2	108.6	C14—C15—H15A	111.2
O1A—C4A—C3A	103.5 (6)	C16—C15—H15A	111.2
O1A—C4A—H4A1	111.1	C14—C15—H15B	111.2
C3A—C4A—H4A1	111.1	C16—C15—H15B	111.2
O1A—C4A—H4A2	111.1	H15A—C15—H15B	109.1
C3A—C4A—H4A2	111.1	O4—C16—C15	106.8 (3)
H4A1—C4A—H4A2	109.0	O4—C16—H16A	110.4
C5—O2—C8	105.1 (2)	C15—C16—H16A	110.4
C5—O2—Yb1	124.6 (2)	O4—C16—H16B	110.4
C8—O2—Yb1	123.9 (2)	C15—C16—H16B	110.4
C13—O4—C16	108.3 (3)	H16A—C16—H16B	108.6
C13—O4—Li1	121.9 (3)		

## References

[bb1] Bruker (2004). *APEX2* and *SAINT* Bruker AXS Inc., Madison, Wisconsin, USA.

[bb2] Chitsaz, S., Iravani, E., Pauls, J. & Neumüller, B. (2001). *Z. Naturforsch. Teil B*, **56**, 759–764.

[bb3] Laugier, J. & Bochu, B. (2003). *LMGP Suite* Suite of programs for the interpretation of X-ray experiments. ENSP/Laboratoire des Matériaux et du Génie Physique, BP 46, 38042 Saint Martin d’Héres, France.

[bb4] McGuinness, D. S., Brown, D. B., Tooze, R. P., Hess, F. M., Dixon, J. T. & Slawin, A. M. Z. (2006). *Organometallics*, **25**, 3605–3610.

[bb5] Mingqing, C., Guang, W., Shanming, Z., Zuen, H., Wenjie, Q. & Wenling, W. (1986). *Wuji Huaxue Xuebao*, **2**, 102–104.

[bb6] Neumüller, B., Hashmatpour, F. & Dehnicke, K. (1996). *Z. Naturforsch. Teil B*, **51**, 602–60044.

[bb7] Sheldrick, G. M. (1996). *SADABS.* University of Göttingen, Germany.

[bb8] Sheldrick, G. M. (2008). *Acta Cryst.* A**64**, 112–122. 10.1107/S0108767307043930 18156677

